# Detection of primary sites in unknown primary tumors using FDG-PET or FDG-PET/CT

**DOI:** 10.1186/1756-0500-4-56

**Published:** 2011-03-09

**Authors:** Jong Sun Park, Jae-Joon Yim, Won Jun Kang, June-Key Chung, Chul-Gyu Yoo, Young Whan Kim, Sung Koo Han, Young-Soo Shim, Sang-Min Lee

**Affiliations:** 1 Division of Pulmonary and Critical Care Medicine, Department of Internal Medicine and Lung Institute of Medical Research Center, Seoul National University College of Medicine, Seoul National University Bundang Hospital, Gyeonggi-do, Korea; 2 Division of Pulmonary and Critical Care Medicine, Department of Internal Medicine and Lung Institute of Medical Research Center, Seoul National University College of Medicine, Seoul, Korea; 3 Department of Nuclear Medicine, Seoul National University College of Medicine, Seoul, Korea

## Abstract

**Background:**

Carcinoma of unknown primary tumors (CUP) is present in 0.5%-9% of all patients with malignant neoplasms; only 20%-27% of primary sites are identified before the patients die. Currently, 18F-fluorodeoxy-glucose positron-emission tomography (18F-FDG PET) or PET combined with computed tomography (PET/CT) is widely used for the diagnosis of CUP. However, the diagnostic yield of the primary site varies. The aim of this study was to determine whether PET or PET/CT has additional advantages over the conventional diagnostic workup in detecting the primary origin of CUP.

**Findings:**

Twenty patients with unknown primary tumors that underwent PET or PET/CT were included in this study. For all patients, the conventional diagnostic workup was unsuccessful in detecting the primary sites. Among 20 patients, 11 had PET scans. The remaining nine patients had PET/CT. In all 20 patients, neither the PET nor PET/CT identified the primary site of the tumor, including six cases with cervical lymph node metastases. The PET and PET/CT revealed sites of FDG uptake other than those associated with known metastases in seven patients, but these findings did not influence patient management or therapy. Two patients had unnecessary invasive diagnostic procedures due to false positive results on the PET or PET/CT.

**Conclusions:**

Although it is inconclusive because of small sample size of the study, the additional value of PET or PET/CT for the detection of primary sites in patients with CUP might be less than expected; especially in patients that have already had extensive conventional diagnostic workups. Further study is needed to confirm this finding.

## Introduction

Carcinoma of unknown primary tumors (CUP) is a biopsy-proven malignancy in which the anatomical origin of the tumor cannot be identified from the patient history, physical examination, laboratory testing, chest radiographs, computed tomography of the chest, abdomen and pelvis, and (in women) mammography [1]. CUP is present in 0.5-9% of patients with malignant neoplasms; however, only 20-27% of primary sites are identified before the patients die [2]. Some studies have reported that although the median survival time of patients with CUP is less than 1 year, if the primary site is identified and specific therapy started, the survival time can be increased [3,4]. However, primary tumors are detected in less than 40% of patients by conventional diagnostic procedures, even when multiple examinations are performed [1].

Currently, positron-emission tomography (PET) with 18F-fluorodeoxyglucose (FDG) or PET combined with computed tomography (PET/CT) is widely used in the diagnostic evaluation of patients with CUP [5]. The rate of detection of the primary site varies; 24.5-41% for the FDG-PET [6,7] and 22-73% with the FDG-PET/CT [8]. These variable diagnostic yields might be due to different patient inclusion criteria and the extent of the diagnostic workup in different studies. Therefore, the efficacy of PET or PET/CT for the detection of primary sites in patients with CUP remains to be determined. Furthermore, there is concern about the false positive PET results in regions endemic for TB [9]. Therefore, the aim of this study was to determine whether PET or PET/CT had additional advantages over conventional diagnostic workups for the detection of the primary origin of CUP.

## Methods

### Patients

The medical records of patients with CUP that underwent PET or PET/CT imaging were reviewed retrospectively. All patients were admitted to the Seoul National University Hospital for further evaluation between January 2003 and September 2005. Carcinoma of unknown primary tumor was defined as a biopsy-proven malignancy whose anatomical origin could not be identified by a conventional diagnostic workup (history, physical examination, laboratory tests, chest radiography, CT of the chest, abdomen and pelvis, MRI of the suspected lesion, endoscopic examinations where indicated, and, in women, mammography). All patients had biopsy-proven malignancies and the results of conventional diagnostic examinations were negative. The workup performed was determined based on the histological results, and therefore, the procedures used to detect the primary sites of tumors differed among the patients.

### PET or PET/CT imaging

All patients underwent whole-body 18F-fluorodeoxy-glucose positron-emission tomography (18F-FDG PET) or PET/CT scans according to the following procedure. Patients were fasted for at least 8 h before receiving an intravenous injection of 555-740 MBq(15-20 mCi; 0.22 mCi/kg body weight) of 18F-FDG. The uptake period was 60-90 min. The PET was performed on a dedicated PET scanner with a 5-min emission acquisition per imaging level. Attenuation correction was performed using the CT technique (140 kV, 80 mA) in the case of the PET/CT. PET images were reconstructed with a 128 × 128 matrix, an ordered subset expectation maximum iterative reconstruction algorithm (six iterations, 16 subsets), a 2-mm Shepp filter and a 16.2-cm field of view. PET/CT images were reconstructed with a 144 × 144 matrix and a 3 D row action maximum likelihood algorithm (two iterations, 0.006 relaxations). The results of PET or PET/CT scans were evaluated by two experienced nuclear medicine physicians that were unaware of the histology of the metastatic sites.

### Evaluation

'Detection of the primary tumor using PET or PET/CT' was defined when additional information about the primary tumor was revealed by PET or PET/CT imaging. Although the suspected primary site was seen on the PET or PET/CT, it was not considered 'detection by PET or PET/CT' if the suspected primary site was seen on other imaging modalities such as the CT. When the FDG uptake site in the PET or PET/CT was confirmed as a benign lesion, this was defined as a 'false positive' PET or PET/CT result.

## Results

Twenty patients (nine men and eleven women) were included in the study. The median age was 54 years and the mean follow-up duration was 26.5 months. Metastases were located in the cervical lymph nodes (*n *= 6), bones (*n *= 4), abdominal lymph nodes (*n *= 3), axillary lymph nodes (*n *= 2), brain (*n *= 1), skin (*n *= 1), omentum (*n *= 1), peritoneum (*n *= 1), and ureters (*n *= 1). The histological findings were distributed as follows: poorly differentiated carcinoma (*n *= 11), adenocarcinoma (*n *= 5), squamous cell carcinoma (*n *= 2), signet ring cell carcinoma (*n *= 1), and leiomyosarcoma (*n *= 1) (Table 1).

**Table 1:** Baseline characteristics of patients included in the study.

Variables	*n *= 20
Sex, male/female	9/11
Age, median (range)	54 (20-74)
Mean follow-up duration (months)	26.5
Site of metastases	
Cervical LN	6
Extracervical LN (abdominal LN, axillary LN)	5 (3, 2)
Bone	4
Brain	1
Others*	4
Pathologic type	
Poorly differentiated carcinoma	11
Adenocarcinoma	4
Squamous cell carcinoma	2
Signet ring cell carcinoma	2
Leiomyosarcoma	1

*skin, mesentery, peritoneum, ureter.

Abbreviation: LN = lymph node

**Figure 1:**
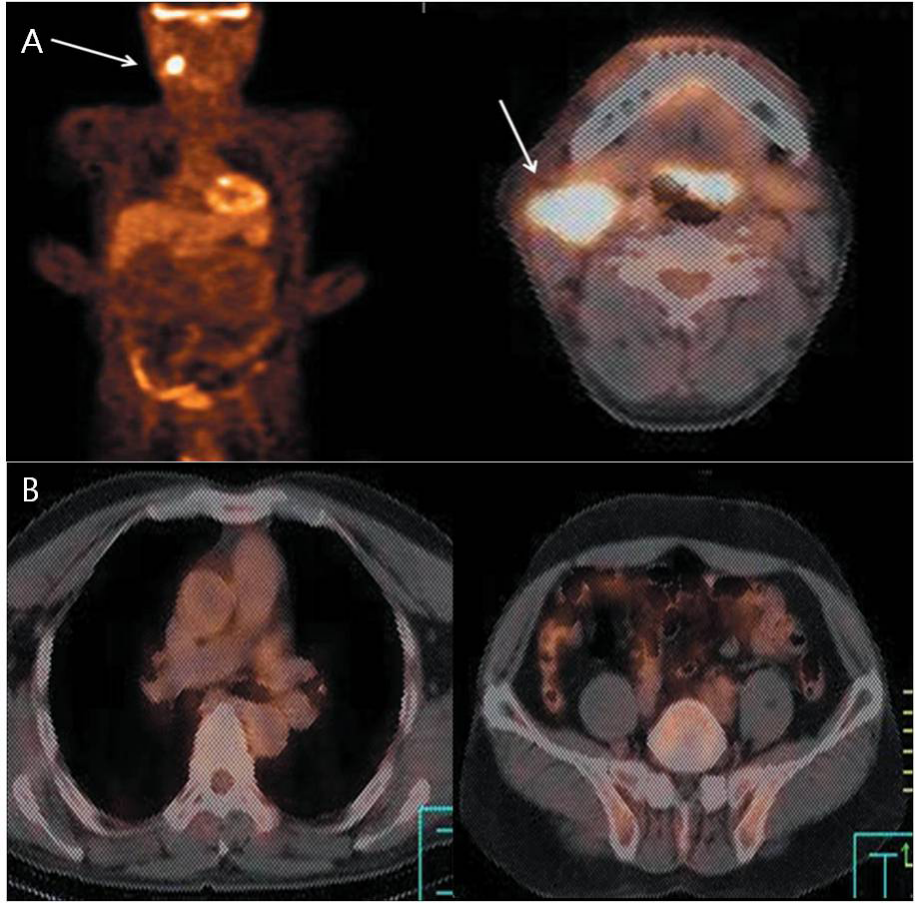
**A 54-year-old male with right cervical lymph node metastasis from an unknown primary tumor (patient 9): The patient had radical neck dissection, tonsillectomy, blind biopsy of the nasopharynx and tongue base**. The pathology revealed metastatic squamous cell carcinoma in one out of 20 lymph nodes. However, there was no evidence of malignancy in other tissues including tonsiles, tongue base, parotid gland, salivary gland, or nasopharynx; (A) PET/CT showed a hypermetabolic lesion (SUV 13.0) in right cervical lymph node(arrow) at level II; (B) There was no additional FDG uptake suggesting a primary site in the transaxial PET/CT scans of the chest and pelvis.

Among the 20 patients, 11 underwent 18F-FDG PET scans, four of whom also underwent PET/CT scans several months after the initial PET scan was performed. The remaining nine out of the 20 patients only had a PET/CT. Data on the 20 patients are shown in Table 2. Neither the PET nor PET/CT detected the putative primary site of the metastatic tumor in any of the 20 patients. In six cases, the initial presentation was cervical lymph node metastasis. Neither the PET nor PET/CT detected the primary site of cervical lymph node metastases. A 54-year-old male (patient no. 9) presented with right cervical lymph node metastasis from an unknown primary tumor. The patient had a radical neck dissection, unilateral tonsillectomy, blind biopsy of the nasopharynx and the tongue base. The pathology revealed metastatic squamous cell carcinoma in one out of 20 cervical lymph nodes. However, there was no evidence of malignancy in the other tissues including tonsil, tongue base, parotid gland, salivary gland, and nasopharynx. The PET/CT showed a hypermetabolic lesion (SUV 13.0) in a right cervical lymph node, at level II. However, there was no additional FDG uptake suggesting a primary site (Figure 1). These findings contrast with those of previous studies [6,10-12] that demonstrated the efficacy of PET for localizing primary sites of cervical lymph node metastases. In this study, not even the PET/CT was able to localize the primary site of the cervical lymph node metastases.

**Table 2:** Results of 20 patients with cancer of unknown primary tumors.

**Patient no**.	Sex	Age	Site of metastases	Pathology	PET or PET/CT	PET or PET/CT finding				Primary site detected by IHC	IHC markers used to detect primary site
								
						Primary tumor	Known metastases	Additional findings	Results of additional findings		
1	M	54	Cervical LN	Squamous cell ca.	PET	not seen	seen	-	-	-	-
2	F	66	Bone	PD	PET/CT	not seen	seen	-	-	-	Vimentin, CK 7, CK 20, CD 68
3	M	55	Cervical LN	PD	PET→PET/CT	not seen	seen	-	-	-	CK 7, CK 19, CK 20, Thyroglobulin,
4	F	57	Bone	Signet ring cell ca.	PET/CT	not seen	seen	Neck, thyroid, hand, forearm uptake	Benign	-	CK 7, CK 20, TTF-1, ER, PR
5	M	56	Skin	Adenocarcinoma	PET→PET/CT	not seen	not seen	Mid-esophagus uptake (subcarinal LN)	Benign	-	CK 7, CK 19, CK 20 TTF-1
6	F	36	Bone	Adenocarcinoma	PET	not seen	seen	-	-	Breast	ER, PR, C-erbB2, CK 7, CK 20, TTF-1, GCDFP-15
7	F	62	Abdominal LN	PD	PET/CT	not seen	seen	Pharynx, thyroid, uptake	Benign	-	-
8	M	15	Cervical LN	PD	PET	not seen	seen	-	-	-	CK, Vimentin, CD 68
9	M	54	Cervical LN	Squamous cell ca.	PET/CT	not seen	seen	-	-	-	-
10	M	46	Brain	Leiomyosarcoma	PET→PET/CT	not seen	seen	Left lower lung uptake	Benign	-	CD 34, Smooth muscle actin, CD 68
11	F	34	Cervical LN	PD	PET	not seen	seen	-	-	Lung	CK 7, CK 20, TTF-1
12	F	60	Omentum	Adenocarcinoma	PET/CT	not seen	*	*	*	-	CK7, CK 20
13	F	48	Peritoneum	Adenocarcinoma	PET/CT	not seen	seen	Inguinal LN uptake	Malignant	-	CK7, CK 20
14	F	60	Axillary LN	PD	PET	not seen	seen	-	-	-	CK 7, CK 20, TTF-1
15	M	61	Cervical LN	PD	PET→PET/CT	not seen	seen	-	-	-	CK, leukocyte common antigen
16	F	53	Ureter	PD	PET/CT	not seen	*	*	*	-	CK 7, CK 20
17	M	59	Abdominal LN	Adenocarcinoma	PET/CT	not seen	seen	Thyroid uptake	Benign	-	CK 7, CK 19, CK 20, TTF-1
18	M	68	Abdominal LN	PD	PET	not seen	*	*	*	-	CD 56, CK
19	F	51	Axillary LN	PD	PET/CT	not seen	seen	-	-	-	CK 7, CK 20, TTF-1
20	F	43	Bone	PD	PET	not seen	seen	Neck LN uptake	Malignant	-	CK 7, CK 20

*PET or PET/CT was done several months later at the time of the initial workup.

Abbreviation: LN = lymph node., ca. = carcinoma, PD = poorly differentiated carcinoma, CT = computed tomography, PET = positron-emission tomography, IHC = immunohistochemistry, ER = estrogen- receptor, PR = progesterone- receptor, CK = cytokeratin, TTF-1 = thyroid transcription factor-1, GCDFP-15 = gross cystic disease

The PET or PET/CT revealed FDG-uptake lesions other than the known metastases in seven out of 20 patients. Five lesions were confirmed as benign (false positive results), and two were pathologically confirmed as another metastatic lesion after biopsy. Three out of five false positive cases (patients no. 4, 7, and 17) also displayed FDG uptake by the thyroid or pharynx (standardized uptake value [SUV] = 2.9-7.1); in patients where the initial physical examinations showed normal thyroids and pharynxes. Clinically, these thyroid glands and pharynxes were not considered to be the primary tumors and did not exhibit malignant changes during the follow-up period.

In two out of five false positive cases, the results of the scans were initially thought to show the primary tumors and a diagnostic workup of these patients was expanded to include invasive procedures. For example, one patient (patient no. 5) with a metastatic adenocarcinoma of the skin had additional FDG uptake around the mid-esophagus (SUV 8.9). To confirm this lesion, the patient underwent a repeat esophagogastroduodenoscopy (Initial result of endoscopy was negative.); however, there was no evidence of a malignancy in the esophagus. A mid-esophagus lesion was observed as subcarinal lymph node uptake on a subsequent chest CT. Because the follow up PET/CT scan showed decreased size and FDG uptake of the subcarinal lymph node, this lesion was confirmed to be a benign lesion. Another patient, with metastatic leiomyosarcoma of the brain (patient no. 10) had mild hypermetabolic findings in the lower left lung field. Because a malignancy could not be ruled out, the patient underwent bronchoscopy and a chest CT. There was no evidence of a malignancy. Because of the false positive PET result, the patient had unnecessary invasive procedures.

In two patients (patient no. 13 and 20) out of the seven that had additional FDG uptake, other metastatic lesions were confirmed by pathological examination of biopsies. Because these metastatic sites were just additional, the management plans of these patients did not change. The results with additional FDG uptake did not positively influence the management and therapeutic plans of the patients.

In four cases, the PET/CT was performed after the initial PET scan (patient no. 3, 5, 10 and 15). The PET/CT, which is anatomically more accurate than the PET, did not confer any additional advantage in the detection of the primary sites of patients with CUP.

## Discussion

Detection of the primary tumor can change the prognosis of patients with CUP by enabling targeted treatment. Previous studies have indicated that PET and PET/CT are useful for the detection of primary sites [7,8,13-15]. In this study, neither the PET nor PET/CT imaging improved the detection of the putative primary sites in patients with CUP that already had thorough conventional diagnostic workups. Neither PET nor PET/CT detected the primary sites in any of the 20 patients with CUP.

Previous studies have reported that PET detects primary lesions in 24%-41% of patients with CUP [7]. However, these studies differ in the definition of patients with CUP and conventional workups. Lassen et al. [16] identified primary cancers in nine out of 20 patients (45%) in their prospective study. Among them, eight patients had primary lung cancers and did not undergo chest CTs during the conventional workups. Bohuslavizki et al. [17] studied 53 patients with CUP, of whom primary tumor sites were detected in 20 (37.8%) using the PET. This study did not include CT or magnetic resonance imaging (MRI) in the conventional workup. Alberini et al. [18] investigated 41 patients with CUP. PET detected primary sites in 26 patients; however, 15 patients had the primary site revealed in the conventional workup. The results of previous studies differed from the findings of this study, which was limited to patients for whom a complete conventional workup, including CT or MRI of suspected lesions, failed to show a primary lesion.

According to the previous literature [2], 20-27% of primary sites are identified before the patients with CUP die. The primary site was not initially identified in all of our study patients and was detected in 2 of 20 cases by immunohistochemical staining of biopsied metastatic lesions during the follow-up period. We included only patients whose primary sites were not identified by initial complete diagnostic workups including CT of chest, abdomen, pelvis and endoscopic examinations. Therefore, the patients in our study might have less chance to detect primary sites.

The poor resolution of PET has been superseded by PET/CT, which identifies anatomical landmarks more accurately. The PET/CT detects the primary tumor in 22-73% of patients with CUP, according to a recent review article [8]. However, in our study PET/CT did not improve the detection rate of primary sites in patients with CUP. The findings are consistent with those reported by Gutzeit et al.[14] that the identification rate of primary cancers using PET/CT was 33%, but the diagnostic accuracy did not differ significantly from that of the other modalities even though it revealed more anatomical detail.

The PET and PET/CT have gained widespread acceptance as useful methods for the management of cancer [19]. However, they do not appear to be effective in identifying a primary lesion after a thorough conventional workup fails to do so. This may be due to the biological characteristics of primary tumors. Primary tumors may disappear after seeding metastases because their angiogenetic incompetence leads to marked apoptosis and cell turnover [20]. Primary tumors that have regressed would not be detected by PET or PET/CT. In this study, neither the PET nor PET/CT detected primary sites in six patients with cervical lymph node metastases, contrary to the findings of other studies [6,12]. The 6 patients with cervical lymph node metastases in this study included 4 poorly differentiated carcinomas and 2 squamous cell carcinomas. The metastases of poorly differentiated carcinoma would have marked cell turnover and apoptosis and that leads to early regression of the primary site. A higher portion of poorly differentiated carcinoma would be one reason for a low detection rate of primary sites in cases with cervical lymph node metastases.

The PET or PET/CT revealed FDG uptake lesions other than the known metastases in seven patients. These additional uptake lesions were of no value for detecting the primary sites of tumors, and false positive FDG uptake lesions complicated the diagnosis. Despite no additional value of the PET or PET/CT in the detection of the primary site, primary lesions were identified in two cases by immunohistochemical staining of biopsied metastatic lesions during the follow-up period (Table 2). Various immunohistochemical markers were used to identify the primary site according to the pathology of the metastatic sites. In one patient (patient no. 6) with metastatic adenocarcinoma, immunohistochemical markers such as the estrogen-receptor, progesterone-receptor, C-erbB2, cytokeratin 7, cytokeratin 20, TTF-1(Thyroid Transcription Factor-1) and GCDFP-15(Gross Cystic Disease Fluid Protein 15) were used to identify the primary site. Immunohistochemical staining for the estrogen-receptor, progesterone-receptor and C-erbB2 were positive, but cytokeratin 7, cytokeratin 20, TTF-1 and GCDFP-15 were negative. Therefore, the primary cancer was presumed to be a breast cancer. Cytokeratin 7, cytokeratin 20 and TTF-1 were used in a patient with cervical lymph node metastasis (patient no. 11). Results of immunohistochemical staining showed positive cytokeratin 7, negative cytokeratin 20 and focal positive TTF-1 in this patient. The primary cancer was presumed to be a non-small cell lung cancer. A careful conventional workup that includes immunohistochemistry would be helpful for cases in which the primary site cannot be successfully identified using PET or PET/CT. PET or PET/CT scans are easy to perform because of their non-invasiveness [5]; however, subsequent invasive procedures and biopsies are inevitable for pathology confirmation of the results of the PET and PET/CT.

The limitations of this study included the following. First, the sample size was small and the study design was retrospective. Second, this study was performed in the early stages of PET and PET/CT, when the PET and PET/CT were not widely used. It is possible that the study results do not reflect current PET or PET/CT scanning.

In conclusion, neither PET nor PET/CT improved the detection of primary sites in patients with CUP in our study. Although it is inconclusive because of small sample size of the study, the additional value of PET or PET/CT for the detection of primary sites in patients with CUP might be less than expected; especially in patients that have already had extensive conventional diagnostic workups. Further study is needed to validate this finding.

## Competing interests

The authors declare that they have no competing interests.

## Authors' contributions

SML had full access to all of the data in the study and takes responsibility for the integrity of the data and the accuracy of the data analysis. JSP contributed to analyzing data and drafting the manuscript. WJK, JKC contributed to collecting and analyzing data. JJY, CGY, YWK, SKH and YSS contributed to making conception and design of this study. All authors read and approved the final manuscript.
